# Prognostic Significance of C-PLAN Index in Patients Treated with Immunotherapy for Non-Small-Cell Lung Cancer

**DOI:** 10.3390/jcm15020642

**Published:** 2026-01-13

**Authors:** Ayse Nuransoy Cengiz, Oktay Bozkurt, Muhammet Cengiz, Metin Ozkan, Mevlude Inanc, Umut Kefeli, Devrim Cabuk, Mustafa Erman, Saadettin Kilickap, Tolga Koseci, Duygu Bayir, Deniz Can Guven, Muslih Urun, Ramazan Cosar, Teoman Sakalar, Nargiz Majidova, Emel Mutlu Ozkan, Cengiz Akosman, Mustafa Ersoy, Elif Sahin, Pervin Can Sanci, Canan Yildiz, Erdem Kolemen, Gözde Agdas, Erkam Kocaaslan, Ezgi Turkoğlu, Sedat Yildirim, Berrak Mermit Ercek, Anıl Karakayali, Hayati Arvas, Mehmet Mutlu Kidi, Sedat Biter, Havva Yesil Cinkir, Latif Karahan, Aslihan Ezgi Apaydin Rollas

**Affiliations:** 1 Department of Medical Oncology Clinic, Faculty of Medicine, Erciyes University, Talas, Kayseri 38039, Turkey; aysenuransoy@hotmail.com (A.N.C.); mhmmtcengiz@hotmail.com (M.C.); metino@erciyes.edu.tr (M.O.); mevludeinanc@hotmail.com (M.I.); 2 Department of Medical Oncology Clinic, Faculty of Medicine, Kocaeli University, İzmit 41060, Turkey; ukefeli@yahoo.com (U.K.); devrimcabuk@yahoo.com (D.C.);; 3 Department of Medical Oncology Clinic, Faculty of Medicine, Hacettepe University, Ankara 06230, Turkey; 4 Department of Medical Oncology Clinic, Faculty of Medicine, Istinye University, Istanbul 34010, Turkey; 5 Department of Medical Oncology Clinic, Faculty of Medicine, Cukurova University Balcalı Hospital, Sarıcam, Adana 01790, Turkey; 6 Department of Medical Oncology Clinic, Faculty of Medicine, Osmangazi University, Eskisehir 26040, Turkey; 7 Department of Medical Oncology Clinic, Faculty of Medicine, Yuzuncu Yıl University, Tusba, Van 65080, Turkey; 8 Department of Medical Oncology Clinic, Faculty of Medicine, Afyonkarahisar Health Sciences University, Afyonkarahisar 03030, Turkey; 9 Department of Medical Oncology, Necip Fazil City Hospital, Kahramanmaras 46050, Turkey; 10 Department of Medical Oncology Clinic, Faculty of Medicine, Vm Medicalpark Maltepe Hospital, Istanbul 34846, Turkey; 11 Department of Medical Oncology Clinic, Faculty of Medicine, Corum Erol Olcok Training and Research Hospital, Corum 19040, Turkey; 12 Department of Medical Oncology Clinic, Faculty of Medicine, Medicalpark Hospital, Antınordu, Ordu 52200, Turkey; 13 Department of Medical Oncology Clinic, Faculty of Medicine, Kutahya City Hospital, Kutahya 43100, Turkey; mustafa.ersoy@ksbu.edu.tr; 14 Department of Medical Oncology Clinic, Faculty of Medicine, Kocaeli City Hospital, İzmit 41060, Turkeyanilozbay@hotmail.com (A.K.); 15 Department of Medical Oncology Clinic, Faculty of Medicine, Marmara University, Pendik, Istanbul 34899, Turkey; erkamkocaaslan@gmail.com; 16 Department of Medical Oncology Clinic, Faculty of Medicine, Kartal Lutfi Kırdar City Hospital, Kartal, Istanbul 34865, Turkey; 17 Department of Medical Oncology Clinic, Faculty of Medicine, Dicle University, Sur, Diyarbakır 21280, Turkey; 18 Department of Medical Oncology Clinic, Faculty of Medicine, Gaziantep University, Sehitkamil, Gaziantep 27310, Turkey; 19 Department of Medical Oncology Clinic, Faculty of Medicine University Hospital Heidelberg, 691120 Heidelberg, Germany

**Keywords:** non-small-cell lung cancer, nivolumab, C-PLAN index, prognosis, survival

## Abstract

**Background/Objectives:** Non-small-cell lung cancer (NSCLC) is a common disease with a high mortality rate and is often treated with immunotherapies; however, prognostic markers are required to identify patients who are most likely to benefit from these treatments. Therefore, we designed this study to assess the prognostic significance of the C-PLAN index, which includes performance status (PS) and C-reactive protein (CRP). **Methods:** A total of 560 patients were included in this multicenter study. Patients had been diagnosed with NSCLC and had received nivolumab therapy. The C-PLAN index, defined in 2022, is a score derived from the combination of PS, CRP, lactate dehydrogenase (LDH), albumin, and neutrophil–lymphocyte ratio (NLR). Patients were classified into good-, moderate-, and poor-prognosis groups according to the C-PLAN score. **Results:** The median metastatic overall survival was 25 months in the group with a C-PLAN score < 2 and 6 months in the group with a C-PLAN score ≥ 2 (*p* < 0.001). The median metastatic progression-free survival was 11 months in the group with a C-PLAN score < 2 and 3 months in the group with a C-PLAN score ≥ 2. **Conclusion:** This is the first comprehensive study demonstrating that the C-PLAN index can be used for prognostic purposes in immunotherapy. This score, which can be easily, economically, and practically calculated in outpatient clinics, can predict patient prognosis and determine who should receive longer durations of immunotherapy.

## 1. Introduction

Lung cancer is the most common lethal cancer worldwide. By 2022, 2,480,675 new cases had been diagnosed. In the same year, 1,817,469 deaths were reported globally. Most patients are diagnosed with NSCLC, which is considered the killer of the modern era [1]. Therefore, intensive research on novel treatment modalities is underway. One of these treatments is immunotherapy, which is increasingly being used, particularly for lung cancer. The basic mechanism underlying this treatment is the cytotoxicity of cancer cells due to T-cell activation [2]. In this new treatment modality, T-cells have a cytotoxic effect on tumor cells. The immune system attempts to destroy cancer cells [3]; this effect is mediated by T-cell receptors. T-cell activation or inhibition, in turn, is mediated by immune checkpoint inhibitors (ICIs). Owing to these receptors, an organism can choose which cells to lyse [4]. Therefore, blockade of the two immune checkpoint receptors, CTLA-4 and PD-(L) 1, is the most effective for clinical cancer therapy [5]. In addition, nivolumab, an anti-PD-1 antibody, has shown favorable responses across many cancer types. As a result of these positive responses, the FDA approved nivolumab for use in non-small-cell lung cancer in 2015 [6]. However, follow-up showed that immunotherapy led to better responses in some patient groups, and objective response rates (ORRs) of ICIs in different types of advanced cancer range from 1% to 85% [7,8,9]. Since identifying the right patient is critical for this new generation of treatments, PD-L1 expression in tumors has been examined. However, this marker is not sufficiently prognostic because of tumor heterogeneity and sampling variability [10]. In fact, anti-PD-1 antibodies may not work sufficiently, even in patients with high levels of PDL-1. Therefore, there is a need for prognostic predictors that enable us to start treatment promptly and with minimal time loss in daily practice [11].

Studies have suggested that individual factors influence ICI clearance. For example, better responses have been observed in patients with low levels of acute-phase reactants, such as neutrophil–lymphocyte ratio (NLR), serum lactate dehydrogenase (LDH), and C-reactive protein (CRP) [12]. In oncology clinics, before initiating ICI therapy in NSCLC patients, the patient’s performance status may be considered a factor in treatment response. However, in these studies, scores were prepared using NLR, platelet-to-lymphocyte ratio, CRP, and LDH levels [13,14,15]. All these scores were aimed at predicting immune system activity, but in clinical practice, performance status (PS) is a prognostic factor [16]. Thus, the C-PLAN index, including the patient’s PS, may be more effective and useful for predicting immunotherapy prognosis [17]. There are two studies in the literature concerning C-PLAN in patients with NSCLC. The first study to define the C-PLAN index found lower survival among patients with a high index score. However, the patients received immunotherapy combined with chemotherapy. Again, the number of patients in this study was insufficient [17]. In another study, only 29 patients received ICIs as monotherapy. The remaining patients received combination therapies and were part of the pooled studies [18].

Therefore, there are no studies on NSCLC patients receiving ICIs alone that can be used to determine which patients respond better to ICIs based on this score. In light of these data, we analyzed the prognostic value of the C-PLAN index, including PS, NLR, albumin, and LDH levels in patients with NSCLC receiving only nivolumab.

## 2. Materials and Methods

Study Design: The study was planned as a multicenter study in our country. These patients had been diagnosed with NSCLC and had received nivolumab therapy. The patient population in our study included those who received treatment for advanced, stage IVB NSCLC between 2015 and 2024. In our country, nivolumab is covered by health insurance in the second tier. Therefore, most patients in our study received nivolumab as a second-line treatment. Currently, chemotherapy + ICI combinations have also been used in our country since July 2025, but during the period when the study was conducted, ICIs could be used alone as a second-line therapy.

Patients who discontinued follow-up or treatment or were under 18 years of age were excluded from the study. Similarly, patients with hematological, connective tissue, or autoimmune diseases were excluded. Performance scores, treatments received, comorbidities and medications, side effects after ICIs, disease progression times, smoking history, metastasis status and sites, local and systemic treatments, neutrophils, lymphocytes, albumin, LDH, and CRP before nivolumab treatment were analyzed retrospectively using the hospital’s filing system. Our study was not a pooled analysis. It was only planned for patients with NSCLC and patients receiving nivolumab treatment as immunotherapy.

All procedures were in accordance with the ethical standards of the responsible committee on human experimentation (institutional and national) and the Helsinki Declaration of 1975, as revised in 2013. The Erciyes University Medical Sciences Clinical Research Board approved a waiver of informed consent due to the study’s retrospective nature. Ethical approval for this study was provided by the Erciyes University Medical Sciences Clinical Research Board, with protocol number 2025/139.

### 2.1. Data Classification

For patients who received ICIs, the maximum C-PLAN index is 5 points: 1 point if the ECOG PS ≥ 2, 1 point if CRP was high, 1 point if LDH was higher than the normal cutoff value, 1 point if albumin was less than 3.5 g/dL, and 1 point if the NLR was greater than 3. Patients with a total score of 5 points were considered to have a poor prognosis. In summary, patients with a score of 0–1 were determined to have a good prognosis, and those with a score of 2–5 were determined to have a poor prognosis (Table 1) [17].

### 2.2. Statistical Analysis

Statistical analyses were performed using “IBM SPSS Statistics for Windows. Version 25.0 (Statistical Package for the Social Sciences, IBM Corp., Armonk, NY, USA)”. Descriptive statistics are presented as counts and percentages (*n*, %) for categorical variables and as mean ± SD and median (min–max) for continuous variables. Receiver Operating Characteristic (ROC) analyses were performed for exploratory purposes to assess cohort-specific discrimination and consistency with previously published threshold values. According to Altman and Royston (2000) [19], “Data-driven threshold values may differ across populations and should therefore be interpreted with caution and preferably in conjunction with established threshold values [19].” Our approach is a common statistical method; however, prognostic classification is primarily based on predefined threshold values from the original C-PLAN publication. ROC curve analyses were performed to evaluate the discriminatory abilities of the individual parameters. According to generally accepted rules, AUC values approaching or exceeding 0.70 were considered indicative of acceptable discrimination ability. These analyses were intended to provide supplementary information and were not used as the primary basis for prognostic inferences.

Progression-free survival (PFS) was calculated as the time from the start of first-line therapy to the date of progression, in months. Metastatic overall survival (mOS) was calculated as the time from the start date of first-line therapy to the date of death or the last follow-up, in months. The risk factors for PFS and mOS were identified using univariate and multivariate Cox regression analyses. The Kaplan–Meier method was used to estimate PFS and mOS probabilities, and the log-rank test was used for group comparisons.

The hazard ratio (relative risk) was obtained by taking the 95% confidence interval, and a *p*-value of 0.05 was considered statistically significant. Variables significant at the *p* < 0.05 level were included in a multivariate model, and backward stepwise selection was used at the *p* < 0.10 stringency level to determine the independent risk factors. Finally, patients who were still alive at the time of the analysis were censored at their last recorded follow-up. The follow-up time was estimated using the reverse Kaplan–Meier method.

## 3. Results

**Figure 1:**
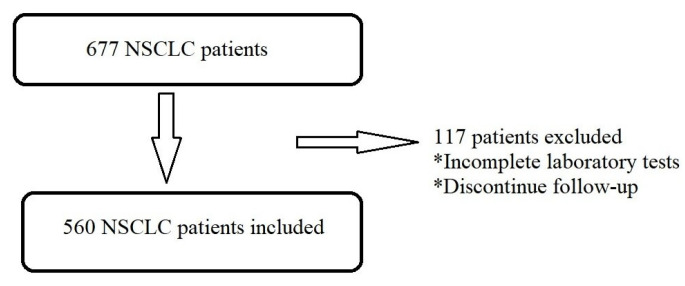
The study population recorded in the registration database.

A total of 677 patients were included in this study. Patients who met the exclusion criteria were excluded from the study, and 560 patients were included (Figure 1). As shown in Table 2, 560 patients were included in this study. The median age of our patients was 63 years, with the youngest patient being 33 years old and the oldest 88 years old. In terms of smoking history, 43.3% of the patients were active smokers, whereas 41.8% were ex-smokers. The mean number of pack-years smoked was 39 ± 20. Most patients received ICI treatment; 80.1% received it in the secondary line, whereas only 4.6% received it in the first line (Table 2).

**Figure 2:**
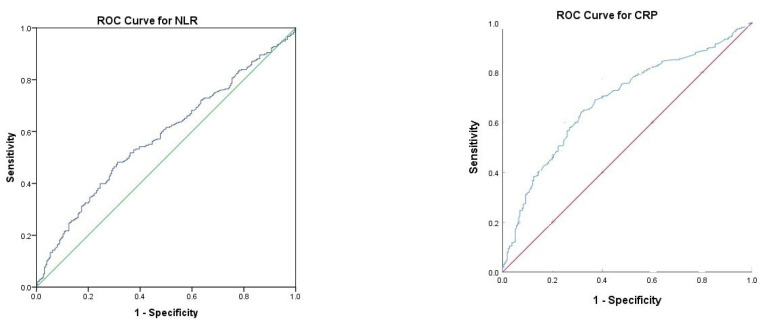
ROC curves of C-PLAN parameters.

According to ROC analysis, NLR (*p* = 0.001) and CRP (*p* < 0.001) were statistically significant in discriminating mortality (Figure 2). According to the ROC analysis, the NLR cut-off value was set at 3.0, and the CRP cut-off value at 16.1 (Table 3).

Univariate analyses of mOS showed significant associations with albumin (*p* < 0.001), CRP (*p* < 0.001), LDH (*p* < 0.001), NLR (*p* = 0.005), PS (*p* < 0.001), and C-PLAN index (*p* < 0.001) (Table 4). Multivariate analysis had prognostic significance for albumin levels (HR 0.74, *p* = 0.02), LDH (HR 0.68, *p* = 0.002), PS (HR 0.69, *p* = 0.01), CRP (HR 0.56, *p* < 0.001), and C-PLAN index (HR 0.65, *p* < 0.001) for mOS (Table 4).

Univariate analyses of mPFS showed significant associations with albumin values (*p* < 0.001), LDH levels (*p* < 0.001), PS (*p* = 0.033), and C-PLAN (*p* < 0.001) (Table 5). Multivariate analysis demonstrated prognostic significance of albumin (HR 0.73, *p* = 0.019), LDH (HR 1.36, *p* = 0.005), and C-PLAN (HR 1.36, *p* = 0.043) for PFS (Table 5).

**Figure 3:**
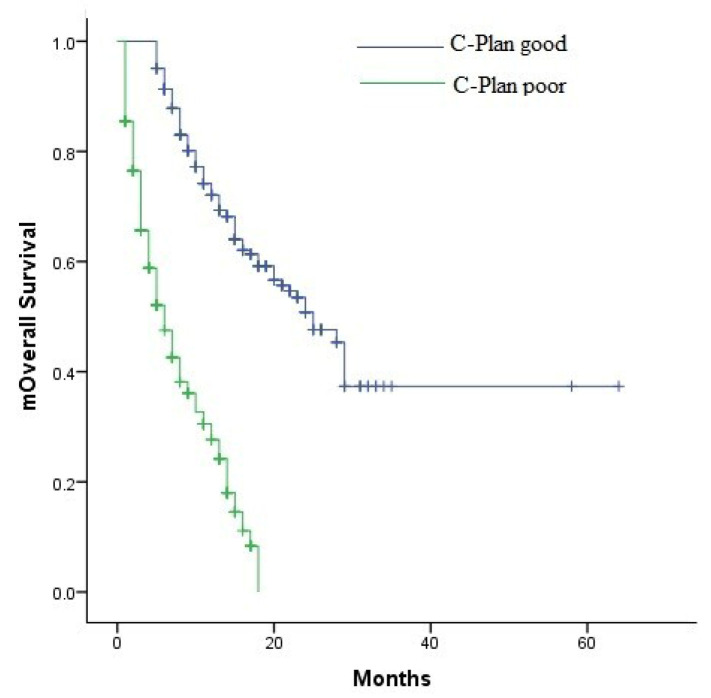
Kaplan–Meier curves of OS according to C-PLAN index groups.

**Figure 4:**
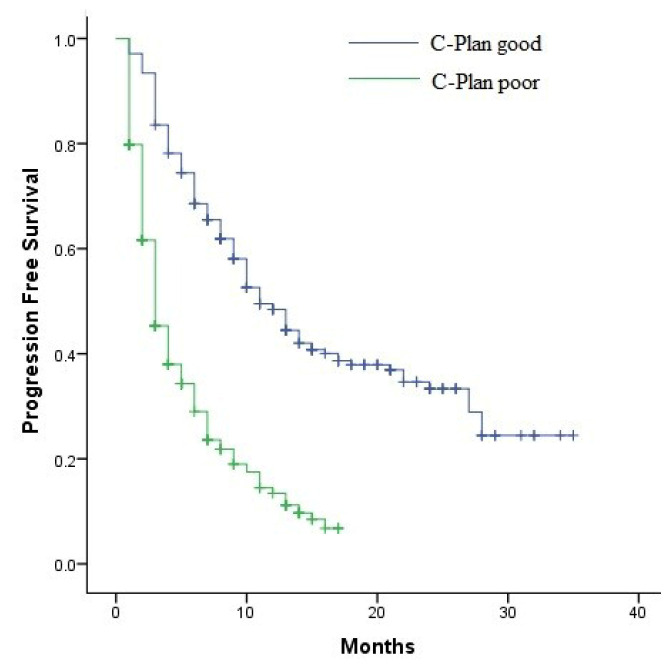
Kaplan–Meier curves of PFS according to C-PLAN index groups.

The median mOS was 25 months (95% CI: 21.1–28.8) in the group with C-PLAN score < 2 and 6 months (95% CI: 4.9–7) in the group with C-PLAN score ≥ 2 (*p* < 0.001) (Figure 3). The median PFS was 11 months (95% confidence interval (CI): 9–12.9 in the group with C-PLAN score < 2 and 3 months (95% CI: 2.5–3.4) in the group with C-PLAN score ≥ 2 (*p* < 0.001) (Figure 4).

## 4. Discussion

Treatment modalities other than chemotherapy are currently being investigated. In particular, studies on ICIs are ongoing. Therefore, it is important to select the correct patient, and PDL-1 levels measured in the peripheral blood may not reflect the tumor microenvironment [20]. Simultaneously, it may take a longer time to access the results of tissue analyses. Therefore, easy, inexpensive, and accessible scores are being studied for clinical practice. In NSCLC patients, the efficacy of nivolumab, independent of PDL-1, has been demonstrated in first-line chemotherapy-treated patients in the Checkmate-57 and 17 studies [21]. Immunotherapy is also used after first-line treatment, particularly in patients who have received first-line chemotherapy or, as in our country, in patients who can receive ICI treatment at least in the second line due to health insurance payment conditions.

Specifically, the tumor microenvironment changes in patients who receive first-line chemotherapy [22]. The tumor becomes more sensitive to the immune system. Therefore, PDL-1 ICIs may not be able to predict the treatment response. However, immune markers may indicate treatment selection and response [23]. Thus, serum inflammatory markers may help predict prognosis [24], including serum NLR and the platelet–lymphocyte ratio. Studies have shown that patients with an NLR of <3 have better survival [25]. Therefore, scoring systems that include parameters such as inflammatory and nutritional indices and the platelet-to-lymphocyte ratio have been developed [26]. Similarly, survival rates in NSCLC patients with elevated LDH levels were nearly 2.5 times worse. Meta-analyses have been conducted on these parameters [27]. Moreover, studies have examined albumin, another inflammatory marker. Again, decreased albumin levels during inflammation were associated with a significant reduction in survival [28]. Among patients with other inflammatory markers, those with low CRP levels lived longer than those with high CRP levels. While 40.6% of patients with low CRP levels were alive at 5 years, only 27.8% of those with high CRP levels survived. Therefore, CRP levels can be used as a survival indicator. These values may indicate prognosis, as they indicate a “hot” tumor [24]. As another parameter, a small portion of the existing scores included PS [29,30].

However, in clinical practice, patients with better PS are thought to have a better treatment response and prognosis [31]. The CRP value and PS are not used in scores such as the systemic immune–inflammation index, HALP score, prognostic nutritional index, or the lung immune prognostic index [32,33,34]. Therefore, the C-PLAN index is distinct from the other scores. The C-PLAN index was created using these data [17]. In this study, patients with a low C-PLAN index had a better prognosis. The risk of death is reduced, and patients have better survival. The high number of patients in this study further supports our findings; therefore, the C-PLAN index can be considered a prognostic score.

Two previous studies have examined the C-PLAN index in lung cancer. In the first study in which the regimen was defined, including 178 patients with NSCLC, patients received pembrolizumab, nivolumab, or nivolumab + ipilimumab combined with chemotherapy. However, most patients received platinum + pemetrexed + pembrolizumab treatment. Consequently, the number of patients in the ICI group in this study was extremely low, and all immunotherapy agents were included in the study and combined with chemotherapy. Therefore, no conclusion can be drawn regarding the applicability of the C-PLAN score in immunotherapy. Our study included 560 patients with advanced NSCLC and was conducted on a larger cohort. All patients received single-agent nivolumab, independent of PDL-1 status. Therefore, the C-PLAN index provides prognostic information only for patients treated with ICI [17].

The other study was a single-center study involving 192 patients. This study included patients with small-cell lung cancer. Similarly, most patients received combination therapies. Only 29 patients received single-agent immunotherapy [18]. Therefore, this study could not demonstrate the prognostic value of the C-PLAN index for immunotherapy. This was a single-center study with a limited number of patients and included all types of lung cancer. In the current multicenter study, all patients received only nivolumab. Overall, the C-PLAN index may be a helpful prognostic score, even in patients who received immunotherapy in the second stage, and there was a significant difference in both PFS and mOS.

Regarding the limitations of our study, it was planned independently of the patients’ PDL-1 status. However, patients receiving ICI therapy can be evaluated based on their PDL-1 status. Thus, better PFS may be achieved in patients receiving ICI. Another missing part of this study was whether patients received blood, blood products, or enteral nutrition support.

In conclusion, the C-PLAN index, a score including CRP, PS, LDH, albumin, and NLR parameters, demonstrated prognostic significance in a large cohort of patients receiving immunotherapy. Moreover, for patients receiving nivolumab therapy, these parameters are practical, readily and rapidly available, and can effectively predict patient survival.

## Figures and Tables

**Table 1:** C-PLAN index parameters.

Variables	Point 0	Point 1
Performance status	0–1	2
Albumin (g/dL)	≥3.5	<3.5
Neutrophil-to-lymphocyte ratio *	<3	≥3
Lactate dehydrogenase (U/L)	<250	≥250
C-reactive protein (mg/L) *	<16.1	>16.1

* Cut-off values determined by ROC analysis. Total points equal to 0 and 1: good. Total points equal to 2–5: poor.

**Table 2:** Characteristics of participants according to C-Plan.

Variables	C-Plan Group		Total (*n* = 560)	*p*-Value
	Poor *n* = 450 (%)	Good *n* = 110 (%)		
**Gender** Female Male	57 (12.7) 393 (87.3)	19 (17.2) 91 (82.8)	76 (13.1) 484 (86.4)	0.719
**Age (years)** <65 ≥65	236 (52.4) 214 (47.6)	58 (52.7) 51 (47.3)	294 (52.5) 266 (47.5)	0.920
**ECOG performance status** 0–1 2	377 (83.8) 73 (16.2)	110 (100) 0 (0)	487 (86.9) 73 (13.3)	**<0.001**
**Diagnosis** Adenocarcinoma Squamous cell carcinoma Other	181 (39.9) 239 (52.8) 30 (6.2)	51 (46.3) 50 (45.4) 9 (8.1)	232 (41.4) 289 (51.6) 39 (7)	0.559
**Metastatic site** Liver Brain Adrenal Bone	60 (18.9) 68 (21.5) 64 (20.2) 131 (41.3)	40 (16.4) 53 (21.8) 70 (28.8) 87 (35.8)	89 (15.9) 121 (21.6) 134 (23.9) 218 (38.9)	0.72 0.91 **0.03** 0.41
**ICI Treatment Line** 1 2 3 4	19 (4) 363 (81) 55 (12.5) 13 (2.5)	7 (6.3) 86 (78.2) 16 (14.5) 1 (1)	26 (4.6) 449 (80.1) 71 (12.6) 14 (2.5)	0.656
**CRP** ≥16.1 mg/L <16.1 mg/L	262 (58.1) 188 (41.9)	43 (39) 67 (71)	305 (54.5) 255 (45.5)	**<0.001**
**Lactate dehydrogenase (LDH)** <ULN ≥ULN	225 (50.2) 223 (49.8)	95 (95) 15 (5)	320 (57.3) 238 (42.7)	**<0.001**
**Albumin** <3.5 g/dL ≥3.5 g/dL	109 (24.2) 341 (75.8)	0 (0) 110 (100)	109 (19.4) 451 (80.6)	**<0.001**

Values are expressed as n (%). Upper limit of reference range: 250 U/L; ULN: Upper limit of normal; mg/dL: milligrams per liter; ECOG: Eastern Cooperative Oncology Group. Statistically significant *p* value are shown in bold.

**Table 3:** Analysis of the value of various clinical parameters in differentiating mortality.

Variables	AUC	%95 CI	Cut-Off	Sensitivity (%)	Specificity (%)	*p*
NLR	0.580	0.533–0.628	≥3	60.7	51	**0.001**
CRP	0.598	0.555–0.641	≥16.10	58.4	59.0	**<0.001**

AUC: area under the curve; 95% CI: confidence interval. NLR: neutrophil–lymphocyte ratio. CRP: C-reactive protein.

**Table 4:** Univariate and multiple cox regression analysis of variables for mOS.

Variables	Univariate		Multiple	
	HR (95%CI)	*p*-Value	HR (95%CI)	*p*-Value
**Age (years)** ≥65 <65	1.00 0.96 (0.77–1.19)	- 0.71		
**Sex** Male Female	1.00 1.1 (0.81–1.59)	- 0.43		
**Smoke** No Yes	1.00 0.9 (0.66–1.23)	- 0.53		
**Histology** Adenocarcinoma Squamous cell carcinoma Others	1.00 0.92 (0.60–1.41) 1.14 (0.74–1.73)	- 0.18		
**Brain metastasis** Yes No	1.00 0.88 (0.68–1.1)	- 0.34		
**CRP** ≥16.1 mg/L <16.1 mg/L	1.00 0.29 (0.23–0.37)	- **<0.001**	1.00 0.50 (0.37–0.68)	- **<0.001**
**ECOG performance status** 2 0–1	1.00 0.35 (0.27–0.46)	- **<0.001**	1.00 0.58 (0.43–0.77)	- **<0.001**
**Lactate dehydrogenase (LDH) ^a^** ≥ULN <ULN	1.00 0.58 (0.47–0.72)	- **<0.001**	1.00 0.67 (0.52–0.86)	- **0.002**
**Albumin** <3.5 g/dL ≥3.5 g/dL	1.00 0.35 (0.27–0.45)	- **<0.001**	1.00 0.59 (0.45–0.78)	- **<0.001**
**NLR** >3 ≤3	1.00 0.62 (0.49–0.77)	- **<0.001**	1.00 0.79 (0.61–1.0)	- 0.09
**C-Plan** ≥2 0–1	1.00 0.20 (0.15–0.26)	- **<0.001**	1.00 0.48 (0.31–0.72)	- **<0.001**

CI: Confidence interval; HR: Hazard ratio; mOS: Metastatic Overall Survival; ECOG PS: Eastern Cooperative Oncology Group performans durumu; mg/L: milligrams per liter; ^a^: Upper limit of reference range: 250 U/L; ULN: Upper limit of normal; NLR: neutrophil to lymphocyte ratio Statistically significant *p* value are shown in bold.

**Table 5:** Univariate and multiple cox regression analysis of variables for PFS.

Variables	Univariate		Multiple	
	HR (95%CI)	*p*-Value	HR (95%CI)	*p*-Value
**Age (years)** <65 ≥65	1.00 1.1 (0.91–1.3)	- 0.26		
**Smoke** No Yes	1.00 1.0 (0.76–1.32)	- 0.95		
**Sex** Male Female	1.00 0.92 (0.69–1.23)	- 0.59		
**Brain metastasis** Yes No	1.00 0.91 (0.72–1.1)	- 0.45		
**Histology** Adenocarcinoma Squamous cell carcinoma Others	1.00 1.0 (0.84–1.1) 1.09 (0.92–1.28)	- 0.57		
**CRP** ≥16.1 mg/L <16.1 mg/L	1.00 0.41 (0.33–0.51)	- **<0.001**	1.00 0.56 (0.43–0.74)	- **<0.001**
**ECOG performance status** 2 0–1	1.00 0.48 (0.37–0.62)	- **<0.001**	1.00 0.69 (0.51–0.92)	- **0.01**
**Lactate dehydrogenase (LDH) ^a^** ≥ULN <ULN	1.00 0.64 (0.52–0.78)	- **<0.001**	1.00 0.68 (0.54–0.86)	- **0.002**
**Albumin** <3.5 g/dL ≥3.5 g/dL	1.00 0.49 (0.39–0.62)	- **<0.001**	1.00 0.74 (0.57–0.96)	- **0.02**
**NLR** >3 ≤3	1.00 0.75 (0.61–0.91)	- **0.005**	1.00 0.88 (0.69–1.12)	- 0.31
**C-Plan** ≥2 0–1	1.00 0.33 (0.27–0.42)	- **<0.001**	1.00 0.65 (0.45–0.93)	- **0.01**

CI: Confidence interval; HR: Hazard ratio; PFS: Progression Free Survival, ECOG PS: Eastern Cooperative Oncology Group performans durumu; mg/dL: milligrams per liter; ^a^: Upper limit of reference range: 250 U/L; ULN: Upper limit of normal; NLR: neutrophil to lymphocyte ratio Statistically significant *p* value are shown in bold.

## Data Availability

The datasets used and/or analyzed during the current study are available from the corresponding author upon reasonable request.
